# Activation and DNA methylation of PANoptosis in polycystic ovary syndrome

**DOI:** 10.1371/journal.pone.0338342

**Published:** 2026-01-05

**Authors:** Yaguang Han, Yanhua Han, Yuenan Feng, Yuanli Shan, Chang Liu, Kexin Wang, Xiaoke Li, Shidi Zhang, Xiaolin Zhu

**Affiliations:** 1 The First Affiliated Hospital of Heilongjiang University of Traditional Chinese Medicine, Harbin, Heilongjiang, China; 2 Heilongjiang University of Traditional Chinese Medicine, Harbin, Heilongjiang, China; 3 The Second Affiliated Hospital of Heilongjiang University of Traditional Chinese Medicine, Harbin, Heilongjiang, China; 4 Department of Traditional Chinese Medicine, Beijing University Third Hospital, Beijing, China; UFRN: Universidade Federal do Rio Grande do Norte, BRAZIL

## Abstract

**Background:**

Polycystic ovarian syndrome (PCOS) is a reproductive endocrine disorder involving various pathophysiological factors and is currently incurable. This study aimed to explore the potential role of pan-apoptosis in PCOS.

**Methods:**

Differentially expressed genes (DEGs) between PCOS and healthy controls were identified in GSE155489 and GSE10946, and enrichment analysis was performed. The activation of PANoptosis in PCOS was evaluated, PANoptosis-related DEGs were screened, and the receiver operating characteristic (ROC) curve was plotted. The methylation modification of PANoptosis-related DEGs were assessed from differentially methylated probes in GSE80468 dataset. Protein expression was detected using Western blot, and DNA methylation levels were measured using PCR in PCOS patients and healthy controls.

**Results:**

PANoptosis was significantly activated in PCOS. The intersection DEGs in GSE155489 and GSE10946 datasets significantly participated in signaling pathways such as Cell adhesion molecules, B cell receptor signaling pathway, and Rap1 signaling pathway. TNFSF10, IL-18, and CASP2 were differentially expressed in PCOS and may be subject to methylation modification, with the ROC curve suggesting they have diagnostic roles in PCOS. Experimental detection confirmed that TNFSF10 is hypomethylated and highly expressed in PCOS, while CASP2 is hypermethylated and lowly expressed in PCOS.

**Conclusion:**

Through comprehensive analysis of gene expression and DNA methylation data, this study revealed the correlation between PCOS and PANoptosis, providing new insights into the molecular basis of PCOS and offering potential biomarkers for the development of future diagnostic and therapeutic strategies.

## Introduction

Over the past few decades, Polycystic ovary syndrome (PCOS), as a complex endocrine disorder, has attracted widespread attention [1]. It affects approximately 7–12% of women of reproductive age and is one of the leading causes of female infertility [2]. The pathological characteristics of PCOS include polycystic changes in the ovaries, hyperandrogenism, ovulatory dysfunction, and insulin resistance [3]. Although inflammation-induced cellular stress and metabolic abnormalities are well-documented in PCOS, the specific modes of cell death contributing to these pathophysiological changes remain poorly defined.

PANoptosis is an integrated and tightly regulated cell death mechanism that combines the molecular machinery of pyroptosis, apoptosis, and necroptosis into a single signaling platform termed the PANoptosome [4]. Activation of PANoptosis is known to induce massive cytokine release, mitochondrial dysfunction, and ROS accumulation, linking inflammatory and metabolic stress [5,6]. These processes mirror several pathological features of PCOS, including oxidative stress in granulosa cells (GCs), disrupted folliculogenesis, and insulin resistance [7]. Notably, NLRP3, a key inflammasome component that serves as a PANoptosis sensor, has been reported to be activated in PCOS ovaries, promoting IL-1β and IL-18 production and impairing granulosa cell function [8]. Similarly, RIPK1/RIPK3/MLKL and caspase-8 signaling have been implicated in ovarian and metabolic inflammation [9], suggesting that the molecular regulators of PANoptosis might be theoretically involved in PCOS progression. However, the role of PANoptosis in PCOS remains unclear.

It has been shown that epigenetic changes have clinical significance for PCOS [10]. Ovarian DNA hypomethylation in polycystic ovary syndrome rats regulates key genes associated with inflammation, insulin signaling, and glucose metabolism [11]. Abnormal DNA methylation affecting lipid metabolism imbalance may provide new insights into the diagnosis and etiology of PCOS [12]. In preclinical models, drugs that reverse DNA methylation have been confirmed to have potential therapeutic effects on PCOS [13].

Gene expression profiling and DNA methylation analysis are key tools for understanding the molecular mechanisms of complex diseases. This study, by integrating gene expression and DNA methylation analyses along with laboratory validation methods, provides new insights into the molecular pathology of PCOS. By thoroughly exploring the role of PANoptosis in PCOS, this research not only enhances our understanding of the molecular basis of this complex disease but also lays the groundwork for the development of new therapeutic strategies.

## Materials and methods

### Data collection and processing

We obtained three publicly available datasets related to PCOS from the Gene Expression Omnibus (GEO) database. GSE155489 [14] containing RNA-seq profiles of oocytes and cumulus granulosa cells (GCs) from three PCOS patients and three non-PCOS women. GSE10946 [15] containing microarray-based expression data from cumulus cells of 19 PCOS patients and 14 non-PCOS women. GSE80468 [16] containing genome-wide DNA methylation data from whole blood of 30 PCOS patients and 30 non-PCOS women.

For RNA-seq data (GSE155489), the raw FASTQ files were downloaded and subjected to quality control using FastQC (v0.11.9). Gene-level counts were generated using featureCounts (v2.0.3) and normalized using the variance stabilizing transformation (VST) in DESeq2(v1.34.0) [17]. For microarray data (GSE10946), raw CEL files were processed using the Robust Multi-array Average (RMA) method for background correction, quantile normalization, and log2 transformation through the affy (v1.74.0) package. For methylation profiling, the quality of methylation data was evaluated using Illumina GenomeStudio software [18]. The minfi R package [19] was used to process methylation data, including background correction and quantitative standardization. Detection P-values >0.01 were filtered out. The β-values representing the methylation proportion at each CpG site were calculated as:


$$\beta =\frac{M}{M+U+\text{100M}}$$

where M and U represent methylated and unmethylated probe intensities, respectively.

### Differential expression and enrichment analysis

Differential gene expression between PCOS and control samples was identified using limma (v3.52.0) [20]. Significantly differentially expressed genes (DEGs) were selected with P < 0.05. Volcano plots was visualized using ggplot2 (v3.4.2). For differential methylation analysis, we used the MethylKit (v1.26.1) package to compare β-values between groups. CpG sites with P < 0.05 were defined as differentially methylated probes (DMPs). The genomic distribution of DMPs was annotated using IlluminaHumanMethylation450kanno.ilmn12.hg19.

To explore the biological significance of DEGs, we performed pathway enrichment using clusterProfiler (v4.6.0) [21]. KEGG enrichment was conducted via the enrichKEGG function with P < 0.05.

### Assessment of PANoptosis

Gene sets related to PANoptosis were retrieved from the Molecular Signatures Database (MSigDB, v7.5.1). We then performed Gene Set Enrichment Analysis (GSEA) using the fgsea (v1.24.0) package with 1,000 permutations to evaluate the enrichment score of PANoptosis-related genes. Gene Set Variation Analysis (GSVA, v1.44.5) was used to calculate enrichment scores at the individual sample level, allowing comparison of PANoptosis activation between PCOS and control samples via t-test.

The diagnostic performance of candidate PANoptosis-related DEGs was assessed using pROC (v1.18.5) [22]. ROC curves were constructed based on normalized expression values, and the area under the curve (AUC) was calculated to evaluate sensitivity and specificity.

### Sample collection

Women aged between 28 and 45 were recruited from The First Affiliated Hospital of Heilongjiang University of Traditional Chinese Medicine, including 10 patients diagnosed with PCOS and 10 healthy non-PCOS women as a control group. Individuals with genetic diseases, other endocrine disorders, or those currently undergoing related medication treatments were excluded. The study protocol was approved by the Ethics Committee of The First Affiliated Hospital of Heilongjiang University of Traditional Chinese Medicine (No. HZYLLBA2024004) and in accordance with the Declaration of Helsinki, and all participants signed an informed consent form before joining the study. A 5 mL sample of peripheral blood was collected using vacuum blood collection tubes containing anticoagulant for subsequent analysis. The experiment was conducted from December 8, 2023 to October 25, 2024.

### Western blot

Whole blood samples were mixed with an equal volume of PBS, followed by the addition of an equal volume of red blood cell lysis buffer, and gently mixed until homogenous. The mixture was centrifuged at 2000 g for 10 minutes at 4°C to remove lysed red blood cells and cell debris, and the supernatant was collected. Total protein was extracted from the supernatant using a protein extraction kit according to the manufacturer’s instructions. Protein concentration was determined using a BCA protein assay kit (Beyotime, China). Equal amounts of protein samples were loaded onto SDS-PAGE gels for electrophoretic separation. The separated proteins were then transferred from the gel to a PVDF membrane. The membrane was blocked with 5% skim milk at room temperature for 1 hour. It was incubated overnight at 4°C with specific primary antibodies (ABclonal, China), followed by incubation with corresponding HRP-conjugated secondary antibodies (ABclonal) for 1 hour. Detection was performed using an ECL detection reagent (Beyotime). β-actin was used as a loading control, and protein expression was quantitatively analyzed using ImageJ software.

### DNA methylation detection

Total DNA was extracted from blood samples using a DNA extraction kit (Shanghai Sangon). The extracted DNA was treated with a bisulfite sequencing conversion kit (Sigma) to convert unmethylated cytosines to uracil, while leaving methylated cytosines unchanged. Specific primers were designed for the methylated and unmethylated sequences of the target genes. PCR amplification was performed using standard procedures. The PCR products were subjected to gel electrophoresis and visualized by staining to assess the methylation status of the target genes.

### Statistical analysis

Graphpad (v9.3.0) was used to calculate statistical differences. The data is represented as mean ± standard deviation. Student’s *t*-test to analyze two different groups. P value <0.05 is considered statistically significant.

## Results

### Differentially expressed genes

**Fig 1:**
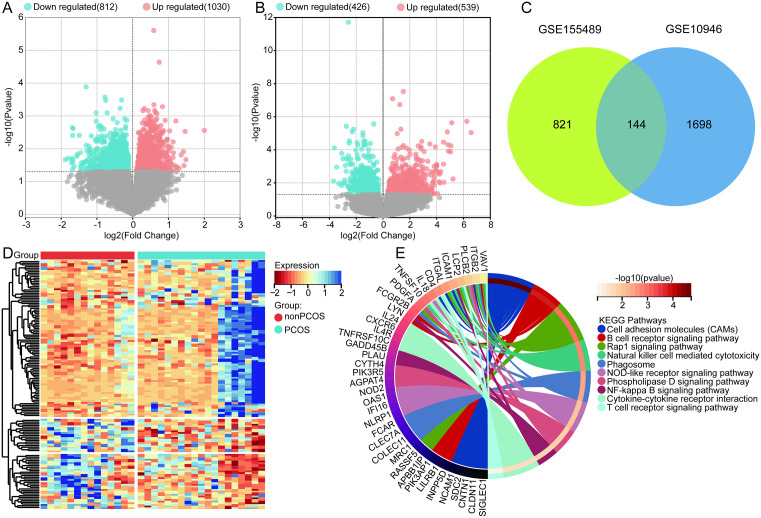
Identification of differentially expressed genes and biological roles. (A) Volcano plot of differentially expressed genes in the GSE10946 dataset. (B) Volcano plot of differentially expressed genes in the GSE155489 dataset. (C) Intersection of differentially expressed genes in GSE10946 and GSE155489 datasets. (D) Heatmap showing the expression of intersection differentially expressed genes. (E) KEGG pathways of intersection differentially expressed genes enrichment.

Through differential analysis between PCOS and controls, we identified 1842 DEGs in the GSE10946 dataset (Fig 1A), and 965 DEGs in the GSE155489 dataset (Fig 1B). Among these, 144 intersection DEGs were found in both datasets (Fig 1C, 1D). Further analysis of these intersection DEGs revealed significant enrichment in key biological functions such as cell adhesion molecules (CAMs), B cell receptor signaling pathway, and Rap1 signaling pathway (Fig 1E).

### PANoptosis in PCOS

**Fig 2:**
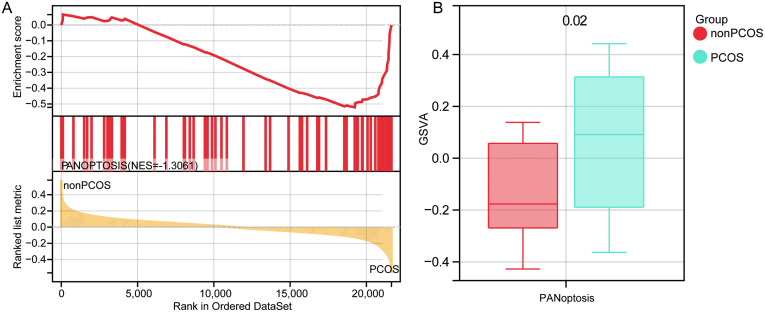
Evaluation of PANoptosis in PCOS. (A) GSEA results of PANoptosis enriched in PCOS. NES, normalized enrichment score. (B) GSVA showing significant activation of PANoptosis in PCOS than controls.

The activation status of PANoptosis in PCOS was investigated through the analysis of the GSE155489 and GSE10946 datasets. GSEA results showed significant enrichment of PANoptosis in PCOS (Fig 2A). Based on the GSVA enrichment analysis, there was a significant difference in PANoptosis between PCOS patients and healthy controls, indicating that PANoptosis is significantly activated in PCOS (Fig 2B).

### Methylation modification of PANoptosis related DEGs

**Fig 3:**
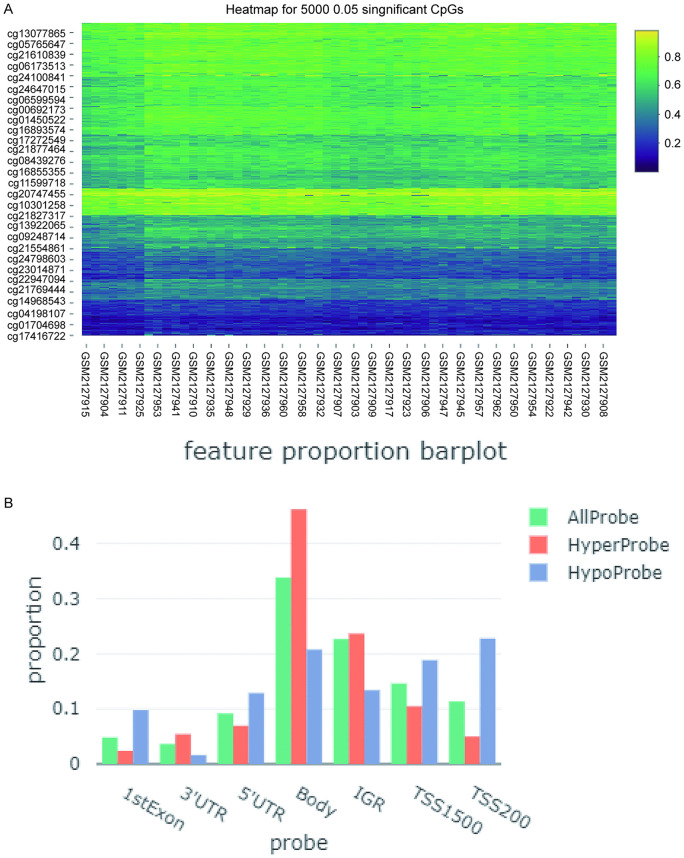
Identification of differentially methylated genes. (A) Heatmap showing top 5000 differentially expressed CpGs between PCOS and controls. (B) Feature proportion of all probes in CpGs.

From the GSE80468 dataset, 17,755 differentially expressed CpGs were identified (Fig 3A), with 14,236 DMPs. Hyperprobes are most common in the gene body across all features, followed by hypoprobes in TSS200 (Fig 3B).

**Fig 4:**
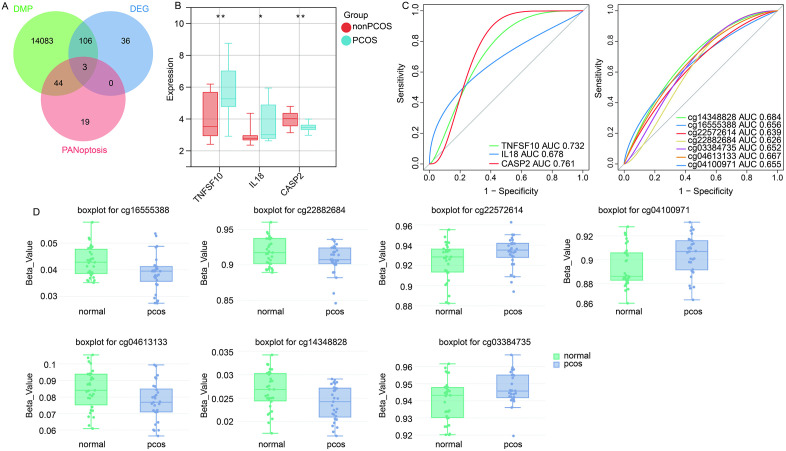
Identification of PANoptosis related DEGs with methylation modification. (A) Intersection of DEGs, DMPs, and PANoptosis related genes. DEG, differentially expressed genes; DMP, differentially methylated probes. (B) Expression of TNFSF10, IL-18, and CASP2 in PCOS and controls in GSE10946 dataset. (C) ROC curves of TNFSF10, IL-18, and CASP2, as well as DMPs. AUC, area under the curve. (D) The β values of DMPs for TNFSF10, IL-18, and CASP2 in PCOS and controls.

Further screening for DMPs related to PANoptosis-associated DEGs revealed three genes (TNFSF10, IL-18, and CASP2), as shown in Fig 4A, Table 1. TNFSF10, and IL-18 were up regulated expression in the PCOS, and CASP2 was down regulated expression (Fig 4B). ROC curves (Fig 4C) showed that the area under the curve (AUC) value of TNFSF10 was 0.732, AUC value of IL-18 was 0.678, AUC value of CASP2 was 0.761. AUC values of DMPs for TNFSF10 (cg16555388, cg22572614, cg22882684), IL-18 (cg04100971), and CASP2 (cg03384735, cg04613133, and cg14348828) were all greater than 0.6. The β values of cg16555388, cg22882684, cg04613133, and cg14348828 were lower in PCOS than controls, and cg22572614, cg04100971, and cg03384735 were higher in PCOS than controls (Fig 4D).

**Table 1:** Summary of PANoptosis-related genes identified in PCOS, including functional roles, methylation patterns, and potential biological significance.

Gene	Expression in PCOS	Methylation pattern	Known/ Proposed function	Potential significance in PCOS
**IL-18**	Upregulated	Hypomethylated	Cytokine activated downstream of NLRP3 inflammasome (1); mediates pyroptosis and inflammation (2)	Promotes chronic low-grade inflammation, disrupts granulosa cell function, contributes to insulin resistance
**TNFSF10**	Upregulated	Hypomethylated	Ligand activating death receptors (3); recruits RIPK1 to form death-inducing signaling complex (4)	Drives inflammatory apoptosis/necroptosis; amplifies immune activation and follicular atresia
**CASP2**	Downregulated	Hypermethylated	Initiator caspase regulating apoptosis and DNA damage response (5)	Reduced CASP2 activity may impair granulosa cell turnover and promote follicular dysfunction

References

1. Barker BR, Taxman DJ, Ting JP. Cross-regulation between the IL-1beta/IL-18 processing inflammasome and other inflammatory cytokines. Curr Opin Immunol. 2011;23(5):591-7.

2. Jorgensen I, Lopez JP, Laufer SA, Miao EA. IL-1beta, IL-18, and eicosanoids promote neutrophil recruitment to pore-induced intracellular traps following pyroptosis. Eur J Immunol. 2016;46(12):2761-6.

3. Teocchi MA, D'Souza-Li L. Apoptosis through Death Receptors in Temporal Lobe Epilepsy-Associated Hippocampal Sclerosis. Mediators Inflamm. 2016;2016:8290562.

4. He W, Wang Q, Xu J, Xu X, Padilla MT, Ren G, et al. Attenuation of TNFSF10/TRAIL-induced apoptosis by an autophagic survival pathway involving TRAF2- and RIPK1/RIP1-mediated MAPK8/JNK activation. Autophagy. 2012;8(12):1811-21.

5. Dorstyn L, Puccini J, Wilson CH, Shalini S, Nicola M, Moore S, et al. Caspase-2 deficiency promotes aberrant DNA-damage response and genetic instability. Cell Death Differ. 2012;19(8):1288-98.

### Molecular experimental verification

**Fig 5:**
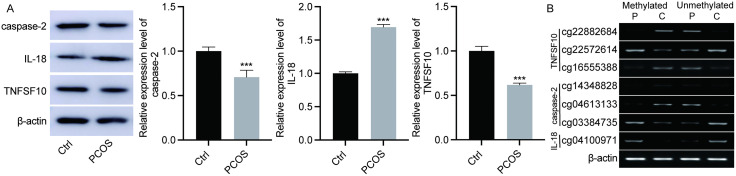
Detection of expression and methylation of TNFSF10, IL18, and CASP2. (A) Protein expression of TNFSF10, IL18, and CASP2 detected by Western blot. (B) Methylation levels of TNFSF10, IL18, and CASP2 detected by PCR. C, controls; P, PCOS.

Through Western blot experiments, we detected significantly higher expression levels of TNFSF10 and IL18 in PCOS patients compared to the healthy control group, while the expression level of CASP2 was significantly reduced (Fig 5A, S1A Fig).

Further PCR detection validated the methylation status of these genes, with cg16555388, cg22882684, cg04613133, and cg14348828 showing hypermethylation in PCOS, and cg22572614, cg04100971, and cg03384735 showing hypomethylation (Fig 5B, S1B Fig).

## Discussion

Due to PCOS being a multifactorial, highly heterogeneous endocrine, metabolic, and psychological disorder, its clinical manifestations can be diverse, leading to long-term and distressing complications for patients [23]. This study utilized public gene expression datasets GSE10946 and GSE155489, along with the methylation dataset GSE80468, to delve into the activation status of PANoptosis in PCOS and its possible molecular mechanisms. In the current research, we revealed that PANoptosis-related genes, modified by methylation in PCOS patients, could have diagnostic implications for PCOS.

Enrichment analysis of DEGs identified in PCOS showed significant enrichment in key biological functions such as CAMs, B cell receptor (BCR) signaling pathway, and Rap1 signaling pathway. Consistent with our study, genes differentially expressed in PCOS were significantly enriched in CAMs [24]. CAMs are essential mediators of cell-cell and cell-matrix interactions within the ovarian microenvironment [25]. Previous studies have demonstrated that abnormal expression of ICAM-1 and VCAM-1 in granulosa cells correlates with increased inflammatory cytokines and impaired folliculogenesis in PCOS patients [26]. Our findings of enriched CAM-related genes therefore suggest that altered adhesion signaling may contribute to aberrant follicular maturation and oocyte quality in PCOS. Enrichment in the BCR signaling pathway points to the involvement of immune dysregulation in PCOS. Hyperactivation of B cells and enhanced BCR-mediated signaling can result in aberrant autoantibody production and chronic low-grade inflammation, both of which are hallmark features of PCOS-associated immune imbalance [27,28]. The Rap1 signaling pathway plays a critical role in the regulation of cell adhesion, oxidative stress response, and insulin signaling. The Rap1 signaling pathway is significantly enriched in PCOS, affecting immune and inflammatory responses, making it particularly impactful on functionality [29]. The enrichment pathways converge on the inflammatory and metabolic dysregulation characteristic of PCOS, future studies combining functional validation of these signaling cascades with in vitro modulation experiments could provide further mechanistic insight into their interplay in PCOS pathogenesis.

The results of GSEA and GSVA both indicated that PANoptosis was significantly activated in PCOS, consistent with previous studies on the role of PANoptosis in other inflammatory and metabolic diseases [30]. The significant activation of PANoptosis in PCOS suggests a central role for cell death pathways in the pathophysiology of PCOS, likely related to local ovarian inflammatory responses and metabolic abnormalities. The activation of the NLRP3 inflammasome directly recruits caspase-1, triggering pyroptosis after cleavage of GSDMD [31], which has been proven to be related to the pathogenesis of PCOS [32]. Activation of the NLRP3 inflammasome in the ovaries is closely related to the dysregulation of GCs and impairs cell proliferation [33]. Studies have shown that apoptosis of ovarian GCs can lead to oocyte apoptosis and follicular atresia, which may be one of the significant factors in the development of PCOS [34,35]. Inflammation can induce necroptosis of GCs, which is crucial in reproductive and endocrine diseases, including PCOS [36]. Necroptosis, a newly recognized type of cell death, has recently been considered a potential factor in the death of GCs in pre-ovulatory follicles [37]. Apoptosis and necroptosis may contribute to luteal regression, affecting follicle development and ovulation [38]. The inflammatory state characterized by decreased antioxidant levels is closely associated with PCOS [39], and oxidative stress damages ovarian tissue, including reduced follicles, apoptosis of granulosa cells, and increased atretic follicles [40]. Our findings offer a new perspective on the pathology of PCOS, suggesting that regulating PANoptosis-related signaling pathways may become a potential strategy for treating PCOS.

Furthermore, we further screened for PANoptosis-related DEGs that are subject to methylation modification, including TNFSF10, IL-18, and CASP2. Through Western blot, we further validated that the expression levels of TNFSF10 and IL18 were significantly higher in PCOS patients compared to the healthy control group, while the expression level of CASP2 was significantly decreased. TNFSF10 is a marker of necroptosis in various diseases [41] and plays a significant promotive role in the development of PCOS. TNFSF10 is significantly related to B cell immune infiltration, and its mediated chronic low-grade inflammation is considered a key factor in the pathogenesis of PCOS [36]. IL-18 is a pro-inflammatory cytokine belonging to the IL-1 superfamily, closely associated with metabolic syndrome and an important predictor of long-term cardiovascular mortality [42]. In PCOS, elevated levels of IL-18 are associated with systemic and visceral obesity as well as insulin resistance [43]. CASP2 has been shown to affect the function of various proteins known to regulate cell proliferation and apoptosis, with CASP2 mRNA expression increasing with the onset of GCs apoptosis [44,45].

Analysis of methylation status revealed a clear correlation between the expression levels of these genes in PCOS and their methylation modifications. TNFSF10 exhibited characteristics of low methylation and high expression, while CASP2 showed high methylation and low expression, suggesting that changes in methylation status might be an important mechanism leading to the abnormal expression of these genes. Our data suggested that TNFSF10, IL-18, and CASP2 in PCOS might be regulated by different methylation sites, and their activity could be related to DNA methylation. AUC values indicated that these genes had certain diagnostic value, a finding supported by the analysis of differentially methylated CpG sites. These results provided new perspectives for understanding the molecular mechanisms of PCOS, while highlighting the significant role of methylation modifications in regulating the expression of PCOS-related genes.

To further elucidate the potential role of PANoptosis in PCOS, it is important to interpret our findings in the context of the hierarchical structure of the PANoptosome. The sensor proteins, such as NLRP3, have shown enhanced in PCOS ovaries, promoting IL-1β and IL-18 maturation and contributing to granulosa cell dysfunction and local inflammation [46]. In our integrated analysis, IL-18, a key downstream mediator of NLRP3 activation, was upregulated and hypomethylated. This supports the notion that inflammasome sensors and their downstream cytokines play an upstream role in driving chronic inflammation in PCOS. Adaptor molecules, including RIPK1, bridge the signaling between sensors and downstream executioners. RIPK1 are known to regulate TNFSF10 (TRAIL)-induced signaling [47]. In our study, TNFSF10 was significantly overexpressed and hypomethylated, suggesting activation of the TNF/TRAIL pathway, which implies that the adaptor-mediated assembly of the PANoptosome may be sensitized in PCOS, contributing to excessive inflammatory cell death in granulosa and immune cells. The effector components, such as CASP2, execute the terminal stages of cell death. In this study, CASP2 was found to be downregulated and hypermethylated, indicating potential transcriptional repression. Reduced CASP2 activity may also disrupt cell cycle regulation and DNA repair, exacerbating ovarian dysfunction [48]. This suggests that epigenetic silencing of key apoptotic effectors may modulate the balance between survival and inflammatory death signaling in PCOS.

Although apoptosis has long been implicated in PCOS, most previous studies have examined it as an isolated cell death pathway within granulosa cells or ovarian tissue [49]. In contrast, our study provides the first integrative evidence that the composite form of programmed cell death, PANoptosis, is significantly activated in PCOS. The concurrent activation of these pathways highlights a more complex and inflammatory form of cell death that could better explain the chronic immune and metabolic disturbances observed in PCOS than apoptosis alone.

## Conclusion

Our study revealed significant activation of PANoptosis in PCOS and identified key regulatory genes associated with it. Especially, TNFSF10, IL18, and CASP2 not only showed significant differences in expression in PCOS, but their expression patterns were closely related to methylation modification, providing new insights for a deeper understanding of the molecular mechanisms of PCOS. These findings provided important information for future research on the molecular mechanisms of PCOS and the development of new therapeutic strategies.

## Supporting information

S1 Fig Detection of expression and methylation of TNFSF10, IL18, and CASP2. (A) The full uncropped Blots image of TNFSF10, IL18, and CASP2 expression detected by Western blot. (B) The full uncropped Gels of Methylation levels of TNFSF10, IL18, and CASP2 detected by PCR. C, controls; P, PCOS.
(PDF)
