# Supplementary material for: Activation and DNA methylation of PANoptosis in polycystic ovary syndrome
Source: PLoS One. 2026 Jan 5;21(1):e0338342. doi: 10.1371/journal.pone.0338342 (PMC12768346; doi:10.1371/journal.pone.0338342)
Supplement: S1 Fig — (A) The full uncropped Blots image of TNFSF10, IL18, and CASP2 expression detected by Western blot. (B) The full uncropped Gels of Methylation levels of TNFSF10, IL18, and CASP2 detected by PCR. C, controls; P, PCOS. (PDF) [file pone.0338342.s001.pdf]

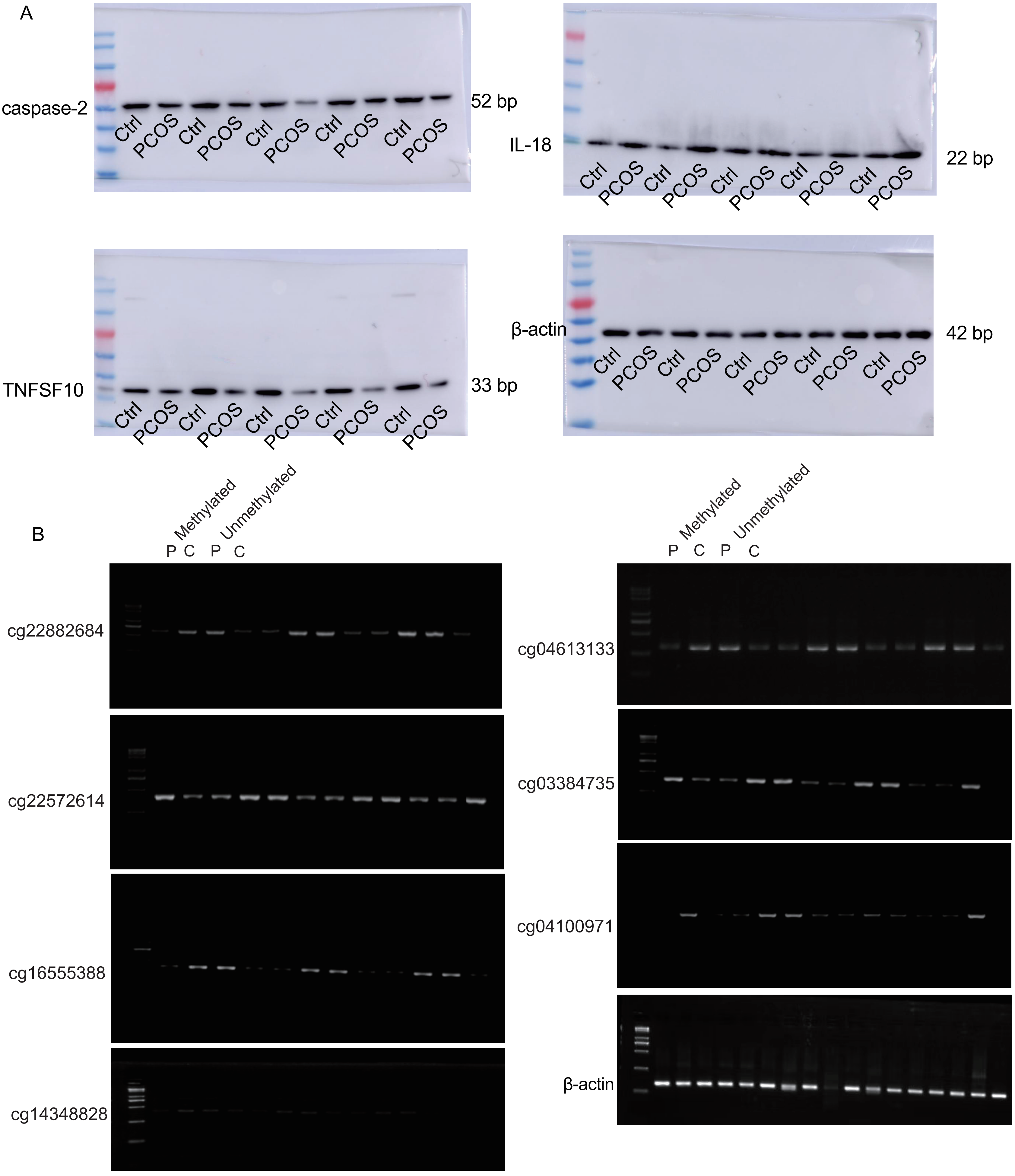

Figure S1. Detection of expression and methylation of TNFSF10, IL18, and CASP2.
(A) The full uncropped Blots image of TNFSF10, IL18, and CASP2 expression detected by Western blot.
(B) The full uncropped Gels of Methylation levels of TNFSF10, IL18, and CASP2 detected by PCR.
C, controls; P, PCOS.
